# Differential Gene Expression in Fusarium Head Blight Pathogens Facilitates Root Infection of Wheat, Maize, and Soybean

**DOI:** 10.3390/plants14162458

**Published:** 2025-08-08

**Authors:** Rukun Li, Huahao Sun, Huilin He, Xinyao Cheng, Mei Deng, Qiantao Jiang, Qiang Xu, Yuming Wei, Yazhou Zhang

**Affiliations:** 1 Triticeae Research Institute, Sichuan Agricultural University, Chengdu 611130, China; lirukun-56@outlook.com (R.L.);; 2 State Key Laboratory of Crop Gene Exploration and Utilization in Southwest China, Sichuan Agricultural University, Chengdu 611130, China

**Keywords:** global warming, cultivation measure, *Fusarium*, transcriptome analysis, cross-host infection

## Abstract

Global food security relies on wheat, maize, and soybean, yet their cultivation faces escalating threats from Fusarium head blight (FHB) pathogens. We demonstrate that agricultural intensification enables cross-kingdom root infections by *Fusarium graminearum* and *F. asiaticum* across these crops. Screening of 180 *Fusarium* strains revealed tripartite host infectivity, with transcriptomics uncovering host-adapted virulence strategies. Transcriptome analysis identified distinct gene expression patterns during the infection of each crop, with *F. graminearum* employing host-specific genes, such as *FgPPDT1* (a pyridoxal phosphate-dependent transferase), for maize root infection. The *FgPPDT1* knockout mutant (Δ*fgppdt1*) exhibited severely impaired root colonization. Our findings establish differential gene expression as a regulatory axis for cross-host adaptation, directly linking FHB transmission risks to wheat–maize intercropping and wheat-soybean rotations.

## 1. Introduction

Fusarium head blight (FHB), a devastating wheat disease, poses a critical threat to global wheat security, with its severity amplified by climate change and shifts in agricultural practices [1]. FHB pathogens are complex and diverse, primarily consisting of *Fusarium asiaticum* and *F. graminearum* [2]. The disease cycle of FHB initiates with overwintering ascocarps on infected plant residues, which disseminate via ascospore production [3]. During the flowering stage, pathogens infiltrate wheat spikes through glume interstices or stomata, subsequently colonizing floral tissues to complete the infection cycle [4]. Following initial colonization, hyphal growth targets stomata and other susceptible sites, ultimately invading the entire wheat ear. This phase culminates in ascospore formation and conidial release, facilitating secondary infections [5,6]. FHB pathogens can remain dormant for up to 10 months within crop debris, while retaining the capacity to cross-infect alternative hosts such as soybean, maize, and rice.

In modern agriculture, wheat–maize intercropping and wheat–soybean rotations are extensively implemented to optimize land productivity and address escalating food demands [7,8]. However, these systems face sustainability challenges due to emerging evidence that retained crop residues elevate Fusarium head blight (FHB) risks. Recent studies confirm that abundant soybean or maize residues significantly increase FHB incidence, prompting recommendations for non-host crop rotations [9,10]. This vulnerability stems from the demonstrated cross-host infectivity of FHB pathogens across both cereals and legumes. Consequently, elucidating mechanisms of pathogen persistence in wheat, maize, and soybean residues—particularly under conservation tillage systems preserving infected debris—is critical for sustainable disease management.

*Fusarium* root rot is a common and widespread disease affecting soybean and maize. Affected plants exhibit impaired emergence and root symptoms, including dark brown lesions (particularly in the lower root system) or complete taproot decay [3,6]. Emerging evidence indicates that FHB pathogens colonize maize and soybean roots, with species such as *F. graminearum* reported from soybean roots in Argentina [9,11,12]. Multi-regional surveys in the United States further identify *F. graminearum*, *F. avenaceum*, and *F. culmorum* as predominant pathogens infecting maize, soybean, and wheat roots [7,13,14]. During early seedling development, these pathogens colonize root tissues, inducing yellowish-brown to dark brown necrotic lesions that reduce emergence rates and compromise crop productivity [12,13,14]. Collectively, these findings suggest FHB pathogens can infect multiple crops through root systems, though direct evidence for cross-host root infection mechanisms remains limited.

In this study, we assessed 180 *Fusarium* strains isolated from symptomatic wheat spikes to evaluate their root colonization across wheat, maize, and soybean hosts. Cross-host infectivity was quantified through reciprocal root inoculation assays. To unravel the molecular basis of host adaptability, we employed RNA sequencing (RNA-seq) to characterize differential gene expression profiles during multi-host infections, with key findings validated through RT-PCR and functional gene knockout experiments. By focusing exclusively on root-mediated infection pathways, this work bridges critical knowledge gaps regarding quantifiable cross-cereal/legume root infectivity and mechanistic studies of host adaptation in root tissues.

## 2. Materials and Methods

### 2.1. Materials and Growing Conditions

All *Fusarium* strains were isolated from wheat spikelets exhibiting visible signs of FHB in our previous study (Table S1) and were cryopreserved at −80 °C in 20% glycerol at Sichuan Agricultural University [2]. For root infection assays, well-characterized cultivars of wheat (*Triticum aestivum* cv. Shumai 482), maize (*Zea mays* cv. Chuandan 99), and soybean (*Glycine max* cv. Nandou 12) commonly grown in Sichuan Province were used as experimental materials. The plants were cultivated under controlled greenhouse conditions (16/8 h light/dark cycle, 70% relative humidity).

### 2.2. Root Infection Test

Seed sterilization and germination were initiated by layering sterile filter paper in Petri dishes, arranging wheat, maize, and soybean seeds in designated positions, saturating the paper with distilled water, and incubating at 25 °C until radicle emergence. Sterile substrate was dispensed into multi-cavity trays (21 cavities; 125 mL volume per cavity) at 60% capacity (*v*/*v*). Fungal inoculum was prepared by homogenizing *Fusarium* colonies (5-day PDA cultures) and blending uniformly with the sterile substrate. Germinated seeds were sown in triplicate groups (3–5 seeds per cavity), with non-inoculated substrate serving as negative controls. Trays were maintained in a greenhouse (16/8 h light/dark cycle; 70% relative humidity) with biweekly irrigation. After 7 days, seedlings were gently uprooted, and their roots were rinsed thoroughly. Infection severity was quantified through fungal biomass analysis. Strains were scored as infectious (‘Infection’) only if root symptoms were observed in both biological replicates, while absence of symptoms in both replicates constituted ‘No infection’. Fungal biomass was quantified via qPCR amplification of *FgGAPDH* (*FGSG_06257*), with calculations following Zhang et al. [15].

### 2.3. RNA Sequencing and Data Processing

Total RNA was extracted from *Fusarium*-infected root tissues using the Plant RNAprep Pure Kit (TIANGEN Biotech, Beijing, China), following the manufacturer’s protocol. RNA sequencing was conducted by E-GENE Technology (Shenzhen, China) on the NovaSeq 6000 platform. Gene expression levels were normalized as Fragments Per Kilobase of transcript per Million (FPKM) mapped reads to account for transcript length and sequencing depth variations [16]. Differential expression analysis was performed using DESeq2 (v1.38.3), with a significance threshold of |log2(fold change)| > 1 and adjusted *p* < 0.01 [17]. Transcript sequences were annotated using the Ensembl Fungi database (release 61—May 2025; http://www.ebi.ac.uk/ena/data/view/GCA_000240135.3, accessed on 5 August 2025). Gene Ontology (GO) enrichment analysis was carried out with TopGO (v3.14.0) to identify functionally enriched terms [18].

### 2.4. Quantitative RT-PCR Validation

Total RNA was isolated from 100 mg (fresh weight) of plant tissue using the E.Z.N.A.™ Total RNA Kit I (Omega Bio-Tek, Norcross, GA, USA), following the manufacturer’s protocol. The RNA was reverse-transcribed into cDNA using the PrimeScript™ RT Reagent Kit with gDNA Eraser (Takara Bio, Dalian, China). Candidate genes for RT-qPCR analysis were selected based on two of three criteria: (1) high expression levels (cycle threshold < 30) detectable by RT-qPCR; (2) the presence of unique genomic regions (>150 bp) for designing species-specific primers; or (3) significant differential expression (|log2(fold change)| > 1, *p* < 0.05) between treatment groups. Relative gene expression was quantified using the 2^−ΔΔCT^ method [19], with *FgGAPDH* (*FGSG_06257*) serving as the endogenous control [15]. The qPCRs were performed using a MyiQ Real-Time PCR Detection System (Bio-Rad, Hercules, CA, USA). All of the primers mentioned above are listed in Table S2.

### 2.5. Construction of Deletion and Complementation Mutants

The target gene sequence was retrieved from the Ensembl Fungi database (http://fungi.ensembl.org). Genomic DNA was isolated from *F. graminearum* mycelia grown on PDA medium for 5 days at 28 °C using the CTAB method [20]. Gene deletion was performed via homologous recombination-based knockout strategies [15]. For targeted gene replacement, the pRF-HU2 vector was introduced into *F. graminearum* through *Agrobacterium tumefaciens*-mediated transformation. Transformants were selected using hygromycin phosphotransferase (HPH) resistance. Complementation constructs were generated by ligating the full-length coding sequence into pCAMBIA1302 (containing the 35S promoter), followed by transformation into deletion mutants. Correct homologous recombination events were verified by PCR amplification, Sanger sequencing of T-DNA flanking regions, and qPCR confirmation of gene deletion according to a previous study [15]. Complementation mutants were validated through RT-PCR and sequencing. All qPCR analyses were conducted on a MyiQ Real-Time PCR Detection System (Bio-Rad, Hercules, CA, USA).

### 2.6. Statistical Analysis

Statistical significance of differences between groups was assessed using Student’s *t*-test in GraphPad Prism 9.0 (GraphPad Software, San Diego, CA, USA). Heatmaps and Venn diagrams were generated with TBtools v2.021 to visualize gene expression patterns and overlapping datasets [21].

## 3. Results

### 3.1. FHB Pathogens Exhibit Multi-Host Root Infectivity

**Figure 1:**
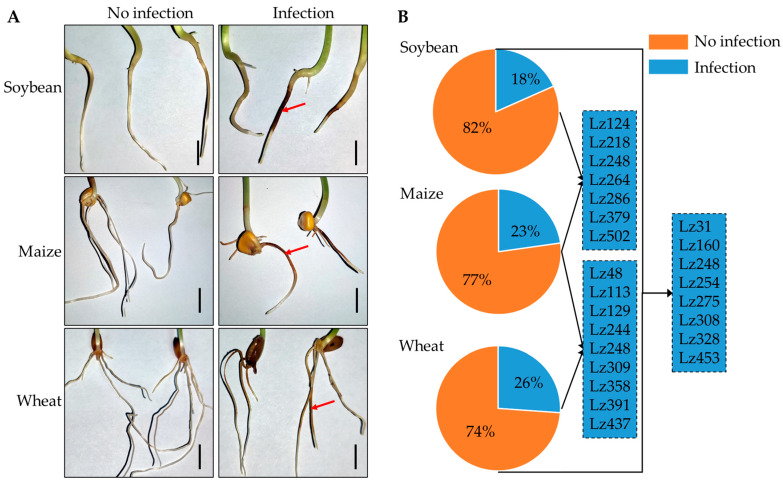
Multi-host root infection by FHB pathogens. (**A**) Pathological manifestations of *Fusarium* infection at 6 day in wheat, maize, and soybean roots. Representative images show necrotic lesions (red arrows) and hyphal infection (scale bar = 2 cm)—5 biological replicates per treatment. (**B**) Statistics on the infection frequencies (%) of 180 *Fusarium* strains (*n* = 3 biological replicates).

To assess the cross-host infection potential of FHB pathogens, 180 *Fusarium* strains were co-inoculated with the root systems of wheat, maize, and soybean (strain details in Table S1). Pathogenicity assays demonstrated successful infection of all three crop roots by FHB pathogens (Figure 1A). Quantitative analysis revealed host-specific infection frequencies: 18% of strains infected soybean roots; 23% colonized maize roots; and 26% exhibited virulence toward wheat roots (Figure 1B; Table S1). Notably, seven strains showed dual infectivity on soybean and maize, nine strains infected both maize and wheat, and eight strains were pathogenic to wheat and soybean. Strikingly, strain Lz248 displayed tripartite infectivity across all three hosts. These results confirm that FHB pathogens can induce soil-borne root diseases in wheat, maize, and soybean, with individual strains capable of infecting multiple hosts.

### 3.2. Host-Specific Gene Expression Drives FHB Pathogenicity

**Figure 2:**
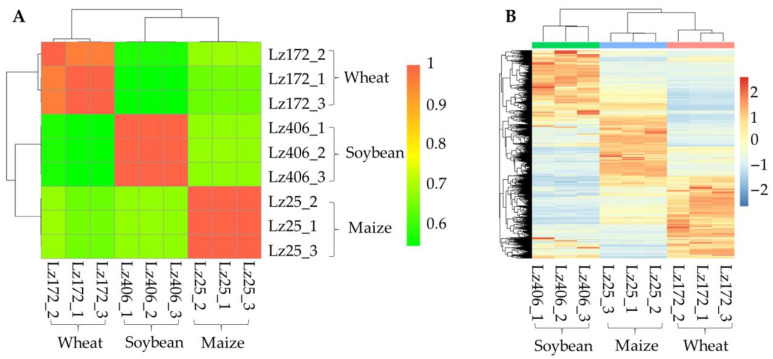
Host-dependent transcriptional regulation in *F. graminearum*. (**A**) Cross-strain transcriptome correlation. Pearson correlation coefficients (r) of gene expression profiles for Lz172, Lz25, and Lz406 during root infection. Dashed lines mark the threshold for significance (r = ±0.5). (**B**) Heatmap of differentially expressed genes. Z-score normalized expression values highlight host-specific transcriptional patterns. Clustering was performed using Euclidean distance and complete linkage.

To investigate the transcriptional mechanisms underlying multi-host infectivity, RNA sequencing was conducted on the roots of wheat, maize, and soybean infected by *F. graminearum* strains Lz172 (wheat-specific), Lz25 (maize-specific), and Lz406 (soybean-specific) (Table S3). These three strains had the same growth rates and phenotypes [6]. Correlation analysis revealed no significant associations (r < 0.3, *p* > 0.05) between the transcriptomes of these strains during host colonization (Figure 2A). Hierarchical clustering further demonstrated divergent gene expression profiles among strains adapted to different hosts (Figure 2B). These findings indicate that host-specific gene expression reprogramming governs the adaptation of *F. graminearum* to the roots of wheat, maize, and soybean.

### 3.3. Host-Specific Transcriptional Reprogramming Underlies F. graminearum Pathogenicity

**Figure 3:**
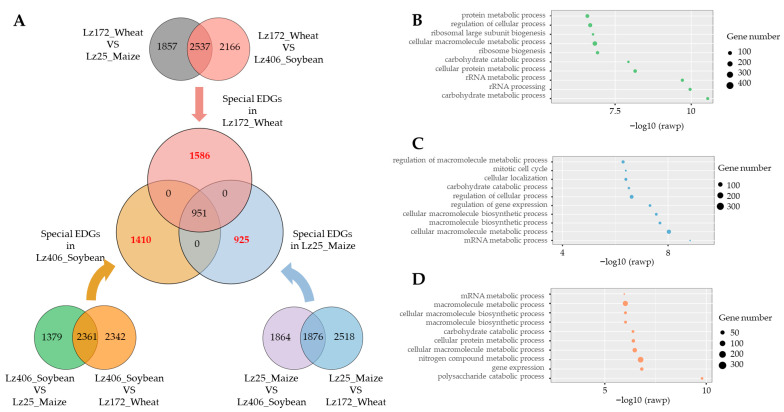
Host-adapted transcriptional networks in *F. graminearum*. (**A**) Host-specific DEG profiling by Venn analysis. Bar plot shows numbers of uniquely upregulated genes during wheat, maize, and soybean root infections. (**B**–**D**) GO enrichment analysis. Bubble charts depict significantly enriched terms (top 10) for wheat (**B**), soybean (**C**), and maize (**D**) infections.

To elucidate host-adaptive gene expression patterns, we identified differentially expressed genes (DEGs) in *F. graminearum* during wheat, maize, and soybean root infections using variance analysis. Strain-specific induction was observed: 1586 DEGs were upregulated during wheat root colonization, 1410 during soybean infection, and 925 during maize pathogenesis (Figure 3A; Table S4). Critically, GO enrichment exposed host-tailored metabolic exploitation: During wheat infection, DEGs enriched in protein metabolic processes and ribosomal large subunit biogenesis suggest accelerated protein synthesis to support invasive growth (Figure 3B). In soybean, DEGs linked to mitotic cell cycle regulation and carbohydrate catabolism indicate enhanced proliferation and carbon utilization from legume-specific substrates (Figure 3C). For maize, DEGs governing mRNA metabolism and cellular macromolecule biosynthesis reflect adaptations to cereal root exudates, potentially enabling amino acid auxotrophy (Figure 3D). These findings demonstrate that *F. graminearum* dynamically modulates its transcriptional landscape to exploit host-specific metabolic niches during root infection.

### 3.4. Host-Specific Gene Induction Drives F. graminearum Adaptation

**Figure 4:**
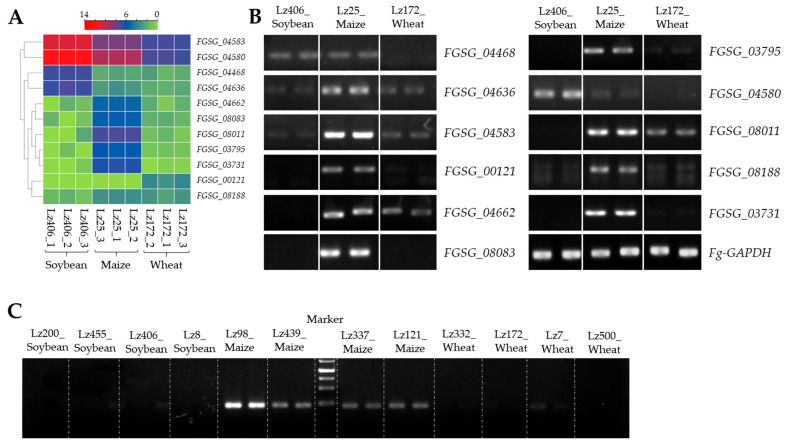
Molecular validation of host-specific virulence genes. (**A**) Heatmap analysis of DEGs identified by RNA-seq during infection of wheat (Lz172), maize (Lz25), and soybean (Lz406) roots. (**B**) RT-qPCR confirmation of host-restricted gene expression. Electropherograms show amplicons for target genes in wheat (Lz172), maize (Lz25), and soybean (Lz406) infections. *FgGAPDH* served as the endogenous control. (**C**) RT-PCR detection of *FgPPDT1* expression across 12 *Fusarium* strains infecting wheat, maize, and soybean roots. All PCR products were verified by sequencing. Data represent two biological replicates per treatment.

To validate transcriptome-derived findings, eleven candidate genes exhibiting host-specific expression patterns were selected for RT-qPCR verification. The analysis confirmed a strict strain-host expression specificity: *FGSG_04468* was exclusively upregulated during infections of soybean and maize roots, while *FGSG_04662* and *FGSG_08011* showed specific induction only during infections of wheat and maize (Figure 4B). Notably, *FGSG_08083* (*FgPPDT1*), encoding a pyridoxal phosphate-dependent transferase, demonstrated maize root-specific expression during *F. graminearum* infection (Figure 4C). These results establish that *F. graminearum* utilizes host-adapted transcriptional programs for root colonization, identifying *FgPPDT1* as a key candidate mediating maize pathogenesis.

### 3.5. FgPPDT1 Is Essential for F. graminearum Virulence in Maize Roots

**Figure 5:**
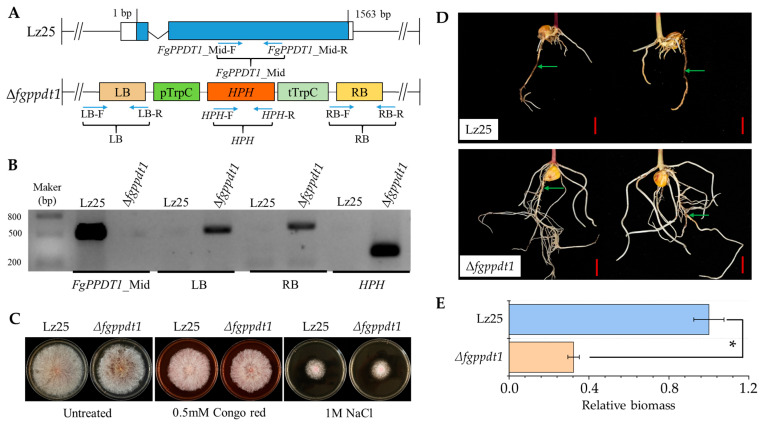
Functional characterization of *FgPPDT1* in maize pathogenesis. (**A**) Schematic representation of the *FgPPDT1* gene structure and validation primer locations (blue arrows) in wild-type strain Lz25 and *FgPPDT1* knockout mutant (Δ*fgppdt1*) mutant. (**B**) PCR verification of ∆*fgppdt1* mutants using primers depicted in (**A**). All primers were listed in Table S2. All PCR products were verified by sequencing. (**C**) Mycelial growth phenotypes of Lz25 and ∆*fgppdt1* strains on PDA plates supplemented with 0.5 mM Congo red or 1 M NaCl at 5 days post-inoculation (dpi). CK: Untreated control. Three biological replicates per treatment. (**D**) Pathogenicity assessment of maize roots inoculated with Lz25 and ∆*fgppdt1* strains at 8 dpi. Necrotic lesions are indicated by green arrows. Scale bar = 1 cm. 5 biological replicates per treatment. (**E**) Quantitative analysis of fungal biomass in maize roots colonized by Lz25 and ∆*fgppdt1* strains at 8 dpi. Data represent mean ± SD from three biological replicates. Asterisks (*) denote statistically significant differences (Student’s *t*-test, * *p* ≤ 0.05).

To elucidate the functional role of *FgPPDT1* in maize root infection, we generated a *FgPPDT1* knockout mutant (Δ*fgppdt1*) in Lz25 strain via homologous recombination (Figure 5A). RT-PCR and qPCR confirmed the absence of FgPPDT1 expression in Δfgppdt1 (Figure 5B and Figure S1). Phenotypic analysis showed no significant differences in mycelial growth between the wild-type Lz25 and Δ*fgppdt1* strains cultured on PDA medium, with neither Congo red nor NaCl stress exhibiting measurable inhibitory effects on either strain (Figure 5C). However, pathogenicity assays demonstrated that Δ*fgppdt1* mutants displayed significantly attenuated virulence and impaired root growth inhibition in maize, as evidenced by markedly reduced necrotic lesion formation compared to infections induced by the Lz25 strain (Figure 5D). Quantification of fungal biomass through qPCR confirmed this phenotypic attenuation, showing 67% reduction in Δ*fgppdt1* infection roots (Figure 5E). We further attempted to create complementation mutants, but all our attempts failed. Despite unsuccessful complementation attempts, these results unambiguously establish *FgPPDT1* as a critical virulence determinant during *F. graminearum* root infection.

## 4. Discussion

The escalating frequency and severity of FHB outbreaks, exacerbated by global warming, underscore the adaptive prowess of *F. graminearum* and *F. asiaticum*. These pathogens leverage their genetic diversity to thrive across diverse agro-climatic conditions [2,22]. Furthermore, cropping systems involving wheat intercropping or rotation influence the predominant *Fusarium* species distributions [23]. In this study, our transcriptome data revealed transcriptional plasticity as the primary adaptive mechanism (Figure 2A,B). GO enrichment analysis indicated that the differentially expressed genes specifically induced during infection of wheat, maize, and soybean roots were all enriched in the “cellular macromolecule metabolic process” (Figure 3B–D). Recent studies suggest that this process represents an important pathway involved in fungal pathogenicity and host adaptability [24,25]. Therefore, these pathogens likely exploit host-specific root exudates and circumvent plant defenses through dynamic gene expression reprogramming, enabling colonization across taxonomically distinct crops.

Pyridoxal phosphate (PLP)-dependent transferases are essential enzymes for amino acid biosynthesis and metabolism across diverse biological systems [26]. Consistent with this functional conservation, the *Mycobacterium tuberculosis* PLP-dependent transferase *Rv2231c* regulates histidine-mediated nitrogen metabolism, a process critical for host survival, proliferation, and pathogenicity [27]. Extending these findings to plant pathology, we identified *FgPPDT1*—a PLP-dependent transferase-encoding gene—as a maize-specific virulence determinant in *F. graminearum* (Figure 4B and Figure 5D,E). We propose it facilitates two key pathogenesis mechanisms: cell wall degradation and pH adaptation, both previously implicated in fungal virulence within acidic niches [28,29]. Phenotypic analysis revealed comparable stress tolerance between wild-type Lz25 and Δ*fgppdt1* strains under Congo red and NaCl exposure (Figure 5C). While dispensable for saprophytic growth and abiotic stress response, *FgPPDT1* is indispensable for maize root colonization, as demonstrated by Δ*fgppdt1*’s severely impaired infectivity (Figure 5D,E). Complementation attempts failed, potentially due to constitutive *FgPPDT1* overexpression driven by the 35S promoter disrupting fungal growth homeostasis. This host-specific requirement reflects adaptation to maize roots’ unique biochemical microenvironment, suggesting *FgPPDT1* mediates metabolic compatibility essential for cross-kingdom adaptation.

Wheat–maize/soybean intercropping and rotation systems, widely adopted in Argentina, China, and the United States to enhance productivity, inadvertently promote *Fusarium* persistence [8,14]. Crop residues act as reservoirs, with wheat stubble inoculating subsequent maize/soybean crops, and vice versa [11,12]. For instance, slower degradation of maize residues under low-temperature conditions elevates the risk of Fusarium head blight (FHB) outbreaks in North America and Europe compared to subtropical regions [10,30,31]. Although most studies confirm that maize or soybean residues increase FHB outbreak risks, few reports indicate that FHB pathogens (*Fusarium* spp.) possess multi-host infectivity. Barros et al. [11,12] isolated *F. graminearum* from soybean flowers, pods, and seeds in Argentina. Similarly, Chang et al. [8] identified *F. graminearum* as the most aggressive species toward soybean. Our study further expands the known host range of these pathogens, demonstrating their capacity to infect wheat, maize, and soybean roots (Table S1). Consequently, recurrent FHB outbreaks likely amplify field inoculum, establishing a feedback loop that jeopardizes maize and soybean yields. To disrupt this cycle, future work should evaluate whether elevated FHB risk correlates with in-field maize or soybean residues using pathogen tracking markers [9,10]. Additionally, three preventive measures are proposed: First, the promotion of resistant crop varieties should be prioritized. Second, pesticides should be applied in a timely manner, taking into account the climatic conditions conducive to the occurrence of FHB. Finally, the burning or conversion of infected straw into biochar can reduce the survival of Fusarium and mitigate the risks of cross-contamination in intercropped and rotated fields [32].

## 5. Conclusions

This study reveals that FHB pathogens exhibit multi-host infectivity, colonizing wheat, maize, and soybean roots. Cross-host adaptation is driven by transcriptional plasticity. Pathogen-specific gene expression patterns were identified, including maize-root virulence mediated by *FgPPDT1*, a pyridoxal phosphate-dependent transferase. Agricultural practices like intercropping and rotation inadvertently promote FHB persistence through crop residue transmission. Mitigation requires integrated strategies such as residue management and resistant cultivars. These findings advance understanding of fungal adaptability and inform sustainable crop protection in a changing climate.

## Data Availability

The transcriptomic datasets generated and analyzed during the current study have been deposited in the NCBI Sequence Read Archive (SRA) and are publicly available at the following link: https://www.ncbi.nlm.nih.gov/bioproject/PRJNA1293538/, accessed on 19 July 2025. All other relevant data are included within the article and its Supplementary Materials.

## Supplementary Materials

The following supporting information can be downloaded at: https://www.mdpi.com/article/10.3390/plants14162458/s1. Figure S1: Verification of ∆*fgppdt1* mutants; Table S1: Infection of various pathogenic strains of Fusarium Head Blight on the roots of wheat, maize, and soybeans; Table S2: Details of the primers used in this study; Table S3: The gene expression of *F. graminearum* during its infection of soybean roots, as analyzed through RNA sequencing; Table S4: List of significantly differentially expressed genes.
