# Supplementary material for: Differential Gene Expression in Fusarium Head Blight Pathogens Facilitates Root Infection of Wheat, Maize, and Soybean
Source: Plants (Basel). 2025 Aug 8;14(16):2458. doi: 10.3390/plants14162458 (PMC12389126; doi:10.3390/plants14162458)
Supplement: Supplementary file 1 [file plants-14-02458-s001.zip › Supplementary Figure S1.pdf]

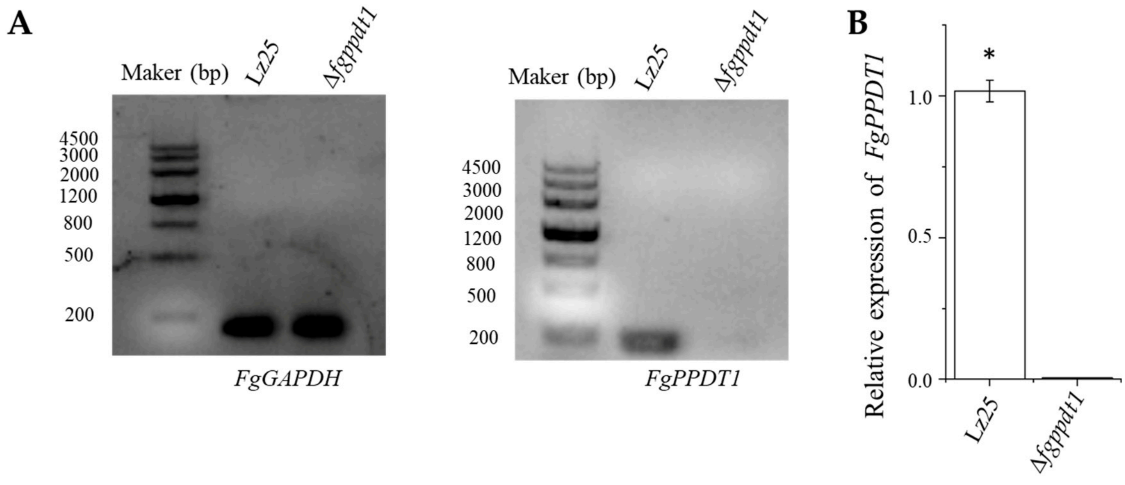

Supplementary Figure S1. Verification of Δ*fgppdt1* mutants. (A) The expression of the *FgPPDT1* was detected in Lz25 and Δ*fgppdt1* mutants by RT-PCR. (B) Relative expression of *FgPPDT1* in Lz25 and Δ*fgppdt1* mutants. Values are average ± standard deviation of three biological replicates per treatment. '*' above each box indicate significant difference ($P < 0.05$).
